# Australian Aboriginal Otitis-Prone Children Produce High-Quality Serum IgG to Putative Nontypeable *Haemophilus influenzae* Vaccine Antigens at Lower Titres Compared to Non-Aboriginal Children

**DOI:** 10.3389/fcimb.2022.767083

**Published:** 2022-04-07

**Authors:** Sharon L. Clark, Elke J. Seppanen, Lea-Ann S. Kirkham, Laura A. Novotny, Lauren O. Bakaletz, Allan W. Cripps, Karli Corscadden, Harvey Coates, Shyan Vijayasekaran, Peter C. Richmond, Ruth B. Thornton

**Affiliations:** ^1^ School of Medicine, The University of Western Australia, Perth, WA, Australia; ^2^ Wesfarmers Centre of Vaccines & Infectious Disease, Telethon Kids Institute, Perth, WA, Australia; ^3^ Centre for Child Health Research, The University of Western Australia, Perth, WA, Australia; ^4^ Centre for Microbial Pathogenesis, Abigail Wexner Research Institute at Nationwide Children’s Hospital, Columbus, OH, United States; ^5^ School of Medicine and Dentistry, Griffith University, Gold Coast, QLD, Australia; ^6^ Perth Children's Hospital (PCH), Perth, WA, Australia

**Keywords:** otitis media, Australian Aboriginal children, IgG, nontypeable *Haemophilus influenzae*, avidity, immunology

## Abstract

**Background:**

Nontypeable *Haemophilus influenzae* (NTHi) is the most common bacterial otopathogen associated with otitis media (OM). NTHi persists in biofilms within the middle ears of children with chronic and recurrent OM. Australian Aboriginal children suffer exceptionally high rates of chronic and recurrent OM compared to non-Aboriginal children. NTHi protein vaccines comprised of antigens associated with both adhesion and persistence in a biofilm are under development and could be beneficial for children with chronic and recurrent OM. Understanding the ontogeny of natural antibody development to these antigens provides insight into the value of vaccinating with particular antigens.

**Methods:**

An in-house multiplex fluorescent bead immunoassay was used to measure serum IgG titres and avidity for three putative vaccine antigens: recombinant soluble PilA (rsPilA), ChimV4, and outer membrane protein 26 (OMP26) in sera from Australian Aboriginal otitis-prone children (n=77), non-Aboriginal otitis-prone children (n=70) and non-otitis-prone children (n=36). Serum IgG titres were adjusted for age, and geometric mean concentrations (GMCs) were compared between groups using a univariate analysis model. Antibody avidity was calculated as a relative avidity index and compared between groups using ANOVA.

**Results:**

Australian Aboriginal otitis-prone children had lower serum IgG titres to rsPilA and ChimV4 than non-Aboriginal otitis-prone children (p<0.001), and non-otitis-prone children (p<0.020). No differences were observed between serum IgG titres from non-Aboriginal otitis-prone children and non-otitis-prone children. There were also no differences in the proportion of high avidity IgG specific for these antigens between these groups. Serum IgG titres to OMP26 were similar between all groups (p>0.670) although otitis-prone children had a higher proportion of high avidity antibodies to this antigen.

**Conclusions:**

Australian Aboriginal otitis-prone children had lower serum IgG titres to 2/3 major NTHi vaccine candidate antigens, suggesting these children are unable to develop persistent IgG responses due to repeated NTHi exposure. These reduced IgG titres may relate to earlier and more frequent exposure to diverse NTHi strains in Aboriginal children through carriage or infection. These data suggest that Aboriginal children may benefit from immunisation with vaccines containing these antigens to increase titres of protective antibodies.

## Introduction

Otitis Media (OM) affects approximately 80% of children by the age of 3 years, making it one of the most common reasons for a child to visit their general practitioner, receive antibiotics and have surgery in industrialised countries (Monasta et al., 2012). One third of children with acute OM develop chronic OM with effusion (lasting ≥3 months) and/or recurrent acute OM (Rovers, 2008); in our paper these children will be referred to as otitis-prone. First nations children are generally more severely affected by OM, including Australian Aboriginal and Torres Strait Islander children, respectfully referred to as Aboriginal from herein, who have one of the highest rates of chronic OM worldwide (Morris et al., 2005). In Aboriginal children, OM starts earlier, is more prolonged, and more severe than in non-Aboriginal Australian children (Williams and Jacobs, 2009; Williams et al., 2009). Most data on OM prevalence in Aboriginal children has been reported from those living in remote and rural areas (Leach et al., 1994; Morris et al., 2005; Leach and Morris, 2007; Wenxing et al., 2012; Leach et al., 2016) however, a recent Western Australian study has shown that urban Aboriginal children also experience disproportionality high rates of OM, with over 50% of 6 month old children suffering from OM (Swift et al., 2020). Nasopharyngeal carriage of otopathogens is a prerequisite for development of bacterial OM (Wenxing et al., 2012). Australian Aboriginal children experience extremely early nasopharyngeal carriage of otopathogens, including nontypeable *Haemophilus influenzae* (NTHi), which is the leading pathogen associated with chronic and recurrent OM (Faden et al., 1991; Leach et al., 1994; Ngo et al., 2016). Recently, we have demonstrated that the presence of NTHi in the middle ear of children at the time of ventilation tube insertion is linked to an increased risk for repeat surgery (Seppanen et al., 2020).

NTHi persists both intracellularly and within polymicrobial biofilms on the middle ear mucosa and in effusion from otitis-prone children (Thornton et al., 2011; Thornton et al., 2013). These biofilms act as infectious reservoirs and enable bacteria to resist host immune responses and traditional antimicrobial therapies (Kania, 2016). NTHi has particular proteins that inititate formation and maintenance of biofilms, including type IV pilus proteins and the DNABII protein family (Jurcisek et al., 2017). Development of vaccines targeting proteins that are important for persistence are likely to be required to both prevent disease, and reduce chronic and recurrent infections in established OM. Other antigens being considered for vaccine development are adhesins that are important in establishing infections such as, Protein D (PD), outer membrane protein P5 (OMP P5), and Protein E (PE), as well as other conserved surface proteins such as, outer membrane protein 26 (OMP26) (Murphy, 2015).

While no NTHi-specific vaccines are licensed, two promising NTHi vaccines containing the PilA protein (the major subunit from the type IV pilus) are in development (Jurcisek et al., 2007; Novotny et al., 2009; Ronander et al., 2009). The first putative vaccine is a multicomponent adjuvanted protein vaccine, NTHi-10-AS01E, which is in phase 3 clinical trials in adults and contains 3 NTHi proteins: recombinant soluble PilA (rsPilA) that is fused with PE, as well as PD and the *Moraxella catarrhalis* antigen Ubiquitous surface protein A2 (UspA2) (Wilkinson et al., 2019). Sera from mice vaccinated with the rsPilA-PE fusion protein prevented NTHi attachment and biofilm formation *in vitro*, as well as initiation of biofilm dispersal resulting in clearance of established biofilm (Ysebaert et al., 2019). Furthermore, passive transfer of sera from chinchillas vaccinated with rsPilA-PE to unvaccinated animals prevented NTHi OM development (Ysebaert et al., 2019). In clinical trials, the NTHi-10-AS01E vaccine increased antibody titres and T-cell responses to all 4 component antigens (Wilkinson et al., 2019). The second NTHi vaccine in development is ChimV4, a chimera of protective epitopes from both rsPilA and OMP P5, which protects against development of NTHi-induced OM and resolves established NTHi biofilm in the chinchilla OM model (Novotny et al., 2013). The potential vaccine antigen, OMP26, has been demonstrated to enhance pulmonary clearance of NTHi in a rat immunisation model (Kyd and Cripps, 1998), but has not advanced to clinical trials.

Vaccines containing NTHi antigens involved in adhesion, biofilm formation and maintenance could be of particular benefit to Australian Aboriginal children who acquire NTHi carriage in early infancy and suffer from high rates of NTHi OM (Leach et al., 1994; Wenxing et al., 2012). Both population and antigen specific differences in natural antibody titres have been demonstrated to exist in otitis-prone children (Pichichero et al., 2010; Kaur et al., 2011a; Kaur et al., 2011b; Wiertsema et al., 2012; Kaur et al., 2016; Thornton et al., 2017a; Thornton et al., 2017b). This was specifically observed for anti-Protein D IgG, where Aboriginal otitis-prone children had significantly lower antibody titres than non-Aboriginal otitis-prone children and non-Aboriginal non-otitis-prone children (Thornton et al., 2017b). In this study, we measured natural antibody titres to putative vaccine antigens rsPilA, ChimV4 and OMP26 in sera from Aboriginal and non-Aboriginal otitis-prone children, as well as non-Aboriginal non-otitis-prone children. We hypothesised that 1) otitis-prone children have reduced antibody titres to NTHi antigens involved in biofilm formation and persistence compared to non-otitis-prone children, and 2) this deficiency will be more pronounced in Aboriginal otitis-prone children, particularly those from rural and remote locations.

## Methods

### Recruitment of Otitis-Prone Children

Children between 1 and 15 years of age were recruited at the time of admission for ventilation tube insertion or tympanoplasty from public hospitals in Perth, Broome, and Derby in Western Australia between 2003 and 2008. Children who met clinical criteria to undergo ventilation tube insertion for either chronic OM with effusion (cOME: middle ear effusion for 3 or more months) or recurrent acute OM (rAOM: 3 or more acute OM episodes in 6 months, or 4 or more episodes in 12 months) or tympanoplasty for chronic perforation of the eardrum following chronic suppurative OM (CSOM: perforation of the tympanic membrane with persistent discharge from the middle ear for 6 or more weeks) were included (Kong and Coates, 2009). Diagnoses were recorded at the time of surgery. Children were identified as Aboriginal or non-Aboriginal based on parental report.

Approval for this study was obtained from the Princess Margaret Hospital for Children (ethics approval number 831EP), Armadale-Kelmscott Memorial Hospital Ethics Committee, Osborne Park Hospital Ethics Committee, the Western Australian Aboriginal Health Ethics Committee, and Aboriginal community-controlled health services in the Kimberley and Eastern Goldfields regions.

### Recruitment of Non-Otitis-Prone Children.

Non-Aboriginal children between 2 and 15 years of age were recruited through the Vaccine Trials Group at the Princess Margaret Hospital for Children (Perth, Western Australia) between 2007 and 2009. Children who were healthy and had no history of recurrent or chronic OM, sinusitis, rhinitis, obstructive sleep disorder, pneumonia or chronic lung disease and no previous ear, nose, or throat surgery were eligible. Approval for this study was obtained from the Princess Margaret Hospital for Children Ethics Committee (ethics approval number 1385EP).

Exclusion criteria for both cohorts included any chromosomal or craniofacial disorders, known immunodeficiency, or receiving immunosuppressive therapy. Written informed consent was obtained from the parents or guardians. Clinical data were collected using parental questionnaires and from medical records.

### Sample Collection

Blood was collected into clot tubes (Vacuette^®^ Greiner Bio-one, Frickenhausen, Germany) from otitis-prone children during surgery, and from non-otitis-prone children during clinic visit. Sera were separated by centrifugation for 10 min at 3200g, aliquoted, and stored at −80°C until analysis.

### Measurement of IgG Titres Specific for NTHi Proteins rsPilA, ChimV4 and OMP26 Using Multiplex Bead-Based Immunoassays

As described previously for PD (Thornton et al., 2017b), the multiplex bead based-immunoassays were modified to include the antigens ChimV4, rsPilA and OMP26. Two final assays were run, the first including OMP26, rsPilA and PD, and the second being a single-plex assay for ChimV4. Modification included testing for cross-reactivity and specificity to determine assay composition. Specificity was determined to be >80% for each antigen. As expected, >80% cross-reactivity was demonstrated between rsPilA and ChimV4, with little cross-reactivity observed with/between other antigens (<10%). All antigens were conjugated to fluorescent carboxylated microspheres for measurement of serum IgG-specific titres (Martinovich et al., 2021). Briefly, samples were diluted 1:200 in serum diluent (phosphate buffer saline, 2% bovine serum and 0.05% tween20). The serum samples were then incubated with 4000 beads per antigen for 30 min at room temperature in the dark, before adding R-phycoerythrin conjugated anti-human IgG (Jackson ImmunoResearch Laboratories, Pennsylvania, USA) and incubated for a further 30 min. The fluorescence of 100 beads within each specific bead region were measured on the BioPlex^®^ 200 System (Biorad). Inter-assay variability was assessed by calculating the percentage coefficient of variation (CV) of the mean concentration of quality control samples (<22% CV). Mean fluorescence intensity (MFI) data was acquired electronically in real-time and analysed using Bio-plex Manager 6.1 software. Data in arbitrary units (AU/mL) were generated from a standard curve of the in-house reference human serum pool and plotted as the geometric mean concentration (GMC) with 95% confidence intervals (CI). Out of range values were repeated using appropriate higher or lower serum dilutions.

### Measuring Avidity of IgG Antibodies Specific for NTHi Proteins PD, rsPilA, ChimV4 and OMP26

Antibody avidity was measured using a modified multiplex bead-based immunoassay. Following the incubation of sera and beads as in the original IgG assay, duplicates of samples were then incubated for 10 min with 25µL of 1M sodium thiocyanate (treatment) or phosphate buffered saline (untreated). R-phycoerythrin conjugated anti-human IgG was added (Jackson ImmunoResearch Laboratories, Pennsylvania, USA) and incubated for a further 30 min and assessed on the BioPlex^®^ 200 System (Biorad). Relative avidity index (RAI) was calculated using the following equation: (MFI of treated sample)/(MFI of untreated sample) x 100. Out of range values were repeated using appropriate higher or lower serum dilutions.

### Statistical Analyses

Host and environmental risk factors were compared between Aboriginal and non-Aboriginal otitis-prone children and non-otitis-prone children using Student’s t-tests for continuous variables (age) and Pearson’s chi-square analyses (asymptotic significant 2-sided P values) for categorical variables (gender, day care attendance, frequencies of diagnoses and surgery). Serum IgG titres were expressed as arbitrary units (AU)/mL against a reference human serum pool and reported as age-adjusted antigen-specific GMCs, with 95% CI. Adjusted GMCs were calculated by using logarithmically transformed, adjusted antibody concentrations. Univariate analyses using a general linear regression model and adjusting for age, were used to assess differences in anti-NTHi antibody titres between Aboriginal and non-Aboriginal otitis-prone children and non-otitis-prone children; and between Aboriginal otitis-prone children from urban and remote areas of Western Australia. RAI were compared between Aboriginal and non-Aboriginal otitis-prone children and non-otitis-prone children using ANOVA; and between Aboriginal children from urban and remote Western Australia using a Mann-Whitney U Test. The IBM SPSS Statistics 26 for Windows software package (IBM, Armonk, NY, USA) was used for all statistical analyses. Data were plotted using GraphPad Prism 9.0 (GraphPad Software Inc., La Jolla, CA, USA).

## Results

### Demographics

A total of 173 children were recruited, including 69 Aboriginal and 72 non-Aboriginal otitis-prone children undergoing surgery for OM, and 32 non-Aboriginal non-otitis-prone children. None of the non-otitis-prone children had any significant history of respiratory infection or OM. For otitis-prone children, 74% of the non-Aboriginal children and 62% of the Aboriginal children were undergoing surgery for ventilation tube insertion, and 19% of the non-Aboriginal children and 33% of the Aboriginal children were undergoing tympanoplasty (**Table 1**). Most non-Aboriginal otitis-prone children were undergoing surgery because of a diagnosis of cOME (76%) and/or rAOM (44%), whereas 62% of the Aboriginal otitis-prone children had a diagnosis of cOME and/or rAOM (25%), and 32% had CSOM (**Table 1**). Significantly more non-Aboriginal otitis-prone children were on antibiotics at the time of surgery and received concurrent adenoidectomies (p=0.015). One Aboriginal otitis-prone child and one non-Aboriginal otitis-prone child were diagnosed with cholesteatoma at the time of surgery. Age was significantly different between Aboriginal, non-Aboriginal otitis-prone children and non-otitis-prone children and was therefore considered a potential confounder and adjusted for in all statistics conducted on normalised IgG titre data.

**Table 1:** Study population.

Characteristic	Non-Aboriginal non-otitis-prone children	Non-Aboriginal otitis-prone children	Aboriginal otitis-prone children	Aboriginal otitis-prone children	
			Total	Urban	Remote
Number [N]	32	72	69	38	31
Age in years [mean (range)] ^+^	8.3	5.2	6.7	5.4	8.2
	(1.6:14.4)	(1.1:13.6)	(1.7:12.7)	(1.7:9.7)	(2.1:12.7)
Female [N (%)]	20 (62)	36 (50)	31 (45)	15 (39)	16 (52)
Had attended day care [N (%)]	18 (56)	40 (56)	27 (39)	16 (42)	11 (35)
Located in remote WA^a^ [N (%)]	0 (0)	2 (3)	31 (45)	0 (0)	31 (100)
History of CSOM^+^ [N (%)]	0 (0)	6 (8)	26 (38)	12 (32)	14 (45)
Current surgery [N (%)]					
Ventilation Tube Insertion	NA	53 (74)	43 (62)	24 (63)	19 (61)
Myringotomy	NA	2 (3)	4 (6)	3 (8)	1 (3)
Tympanoplasty^b^	NA	14 (19)	23 (33)	11 (29)	12 (39)
Adenoidectomy^+^	NA	11 (15)	1 (1)	0 (0)	1 (3)
Principal diagnosis [N (%)]					
OME	NA	55 (76)	43 (62)	25 (66)	18 (58)
rAOM*	NA	32 (44)	17 (25)	12 (32)	5 (16)
CSOM*	NA	4 (6)	22 (32)	7 (18)	15 (48)
Immunisations up to date^c^ [N (%)]	32 (100)	64 (93)	51 (98)	30 (97)	21 (100)
Receiving antibiotics at time of surgery^d+^ [N (%)]	0 (0)	6 (9)	2 (3)	0 (0)	2 (7)

^+^P-value <0.05 between non-otitis-prone, non-Aboriginal and Aboriginal otitis-prone children.

*P-value<0.05 between non-otitis-prone, non-Aboriginal and Aboriginal otitis-prone children, and between Aboriginal otitis-prone children from urban and remote areas.

a Non-otitis-prone children, n = 32; non-Aboriginal otitis-prone children, n = 71; Aboriginal otitis-prone children, n = 69.

b Seven tympanoplasties were performed due to failure of tympanic membrane healing after ventilation tube insertion.

c Non-otitis-prone, n = 32; non-Aboriginal otitis-prone children, n = 69; Aboriginal otitis-prone children, n = 52, Aboriginal otitis-prone children from Urban n=31 and Remote n=21.

d Non-otitis-prone, n = 31; non-Aboriginal otitis-prone children, n = 70; Aboriginal otitis-prone children, n = 65, Aboriginal otitis-prone children from Urban n=37 and Remote n = 28.

NA, not applicable.

### Natural Serum IgG titres for NTHi PilA Derived Proteins Were Significantly Lower in Aboriginal Otitis-Prone Children Compared to Non-Aboriginal Otitis-Prone and Non-Otitis-Prone Children

**Figure 1:**
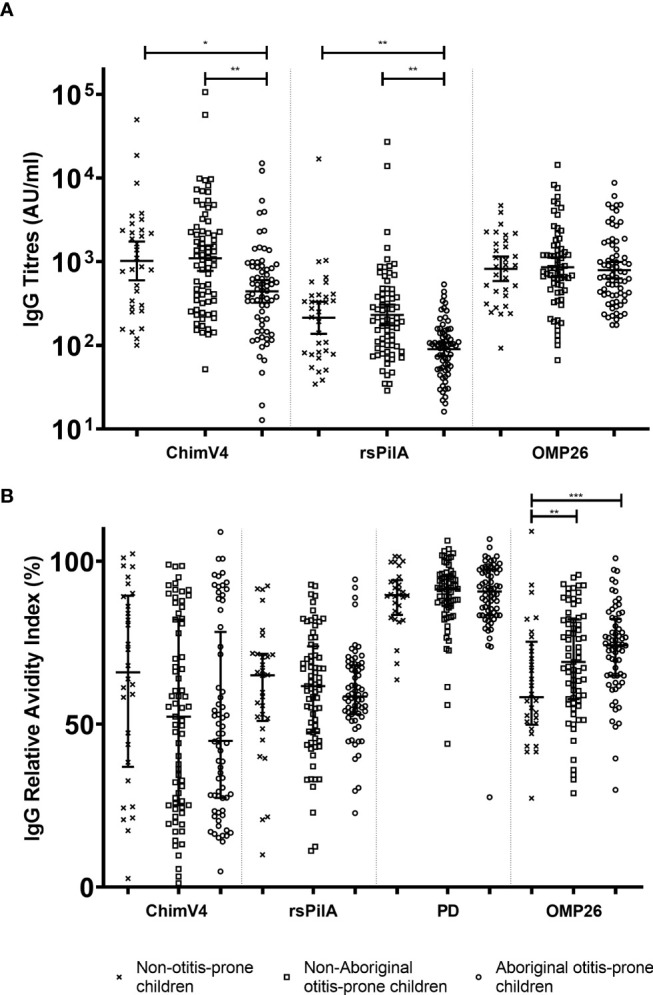
Comparison of serum IgG titres and relative avidity indices between Aboriginal otitis-prone, non-Aboriginal otitis-prone and non-otitis-prone children. **(A)** Comparison of serum IgG titres between study groups. Levels of serum IgG against NTHi proteins are presented for each individual child, with the horizontal bars depicting the GMC ± 95% CI (not age adjusted). Statistical analyses were conducted on the log transformed GMC data, adjusted for age. **(B)** Comparison of serum IgG RAI between study groups. RAI serum IgG against NTHi proteins are presented for each individual child, with the horizontal bars depicting the median ± Interquartile Range. ChimV4, chimeric protein V4 (rsPilA+protein 5), rsPilA, recombinant soluble PilA; PD, Protein D; OMP26, outer membrane protein 26. *P ≤ 0.05; **P ≤ 0.01, ***P ≤ 0.001.

Australian Aboriginal otitis-prone children had lower serum IgG titres to rsPilA and ChimV4 (GMC: 88.94 and 429.66 AU/mL respectively) compared to non-Aboriginal otitis-prone children (GMC: 240.57 and 1210.76 AU/mL respectively), and non-otitis prone children (GMC: 227.69 and 872.18 AU/mL respectively), p<0.05 (**Figure 1A**). There were no significant differences between non-Aboriginal otitis-prone children and non-otitis-prone children. Serum IgG titres specific for OMP26 were similar between groups (GMC: Aboriginal otitis-prone children 1060 AU/mL, non-Aboriginal otitis-prone children 1054 AU/mL and non-otitis-prone children 820.2 AU/mL). Antigen-specific IgG titres were correlated with age to assess IgG development during childhood in each group. Weak correlations were observed between age and NTHi specific IgG titres in each group (R = -0.1-0.33; **Table 2**).

**Table 2:** Correlation of antigen specific IgG titres with age.

Antigen	Group	Correlation Coefficient	P-value
ChimV4	Non-otitis-prone children	0.279	0.123
	Non-Aboriginal otitis-prone children	0.251	0.033
	Aboriginal otitis-prone children	0.079	0.518
rsPilA	Non-otitis-prone children	0.123	0.501
	Non-Aboriginal otitis-prone children	0.257	0.029
	Aboriginal otitis-prone children	-0.093	0.448
OMP26	Non-otitis-prone children	0.078	0.671
	Non-Aboriginal otitis-prone children	-0.059	0.620
	Aboriginal otitis-prone children	0.077	0.531

Serum IgG titres for each antigen were plotted against each other to compare relationships between different antigen-specific IgG titres. There was a strong correlation for IgG titres specific for rsPilA and ChimV4 (R= 0.8; p<0.001) (**Supplementary Figure 1A**). There was also a strong relationship between rsPilA and ChimV4 and previously reported PD titres (R= 0.9 and 0.77 respectively p<0.001) (**Supplementary Figures 1B, C**). Aboriginal otitis-prone children had the lowest IgG titres of all groups for rsPilA, ChimV4 and PD, which were distinct from the other 2 groups (**Supplementary Figures 1A**–**C**). The non-Aboriginal otitis-prone children and non-otitis-prone children had higher IgG titres, which overlapped in distribution. IgG titres specific for rsPilA, ChimV4 and PD had a weak relationship with titres against OMP26 (R <0.4 p<0.001; **Supplementary Figures 1D**–**F**).

### Otitis-Prone Children Produce IgG Against Important Biofilm Proteins With Equivalent Avidity Indices to Non-Otitis-Prone Children

Australian Aboriginal otitis-prone children, non-Aboriginal otitis-prone children, and non-otitis-prone children had similar IgG RAI for rsPilA and PD (median RAI: 59%-62% and 89%-92% respectively). In contrast, the median RAI of non-otitis-prone children for IgG specific for OMP26 (58%) was significantly reduced compared to Aboriginal (74%) and non-Aboriginal (70%) otitis-prone children (**Figure 1B**). Australian Aboriginal and non-Aboriginal otitis-prone children showed a trend towards reduced RAI to ChimV4 compared to non-otitis-prone children (median RAIs 45%, 54% and 63% respectively), however, this did not reach significance (p=0.195). RAIs for ChimV4 and rsPilA showed a moderate correlation (R= 0.5-0.7; p<0.001) for all 3 groups, however, not for other antigens (R=-0.2-0.4). Overall, the RAI to PD was highest compared to other antigens, followed by OMP26, with the RAIs for rsPilA and ChimV4 being the lowest.

Very weak correlations between RAIs and age were observed for all groups and antigens (R=-0.3-0.4) (**Table 3**). Furthermore, only weak relationships between antibody titres and RAI were observed for each antigen (R=-0.2-0.4) (**Table 4**).

**Table 3:** Correlation of antigen specific IgG RAI with age.

Antigen Specific	Group	Correlation Coefficient	P-value
ChimV4	Non-otitis-prone children	-0.337	0.059
	Non-Aboriginal otitis-prone children	0.230	0.052
	Aboriginal otitis-prone children	0.106	0.388
rsPilA	Non-otitis-prone children	0.318	0.076
	Non-Aboriginal otitis-prone children	0.391	0.001
	Aboriginal otitis-prone children	0.046	0.710
Protein D	Non-otitis-prone children	0.168	0.358
	Non-Aboriginal otitis-prone children	0.116	0.333
	Aboriginal otitis-prone children	-0.03	0.808
OMP26	Non-otitis-prone children	0.028	0.880
	Non-Aboriginal otitis-prone children	0.065	0.585
	Aboriginal otitis-prone children	0.110	0.370

**Table 4:** Correlation of antigen specific IgG RAI and IgG titres.

Antigen Specific	Group	Correlation Coefficient	P-value
ChimV4	Non-otitis-prone children	0.110	0.556
	Non-Aboriginal otitis-prone children	0.158	0.184
	Aboriginal otitis-prone children	0.168	0.167
rsPilA	Non-otitis-prone children	0.157	0.399
	Non-Aboriginal otitis-prone children	0.175	0.141
	Aboriginal otitis-prone children	0.361	0.002
Protein D	Non-otitis-prone children	0.345	0.058
	Non-Aboriginal otitis-prone children	-0.040	0.741
	Aboriginal otitis-prone children	-0.157	0.197
OMP26	Non-otitis-prone children	0.217	0.242
	Non-Aboriginal otitis-prone children	0.146	0.220
	Aboriginal otitis-prone children	0.213	0.079

### Australian Aboriginal Otitis-Prone Children From Remote Areas Undergoing Surgery for OM, Have Similar Serum IgG Titres and RAIs to Those From Urban Areas

**Figure 2:**
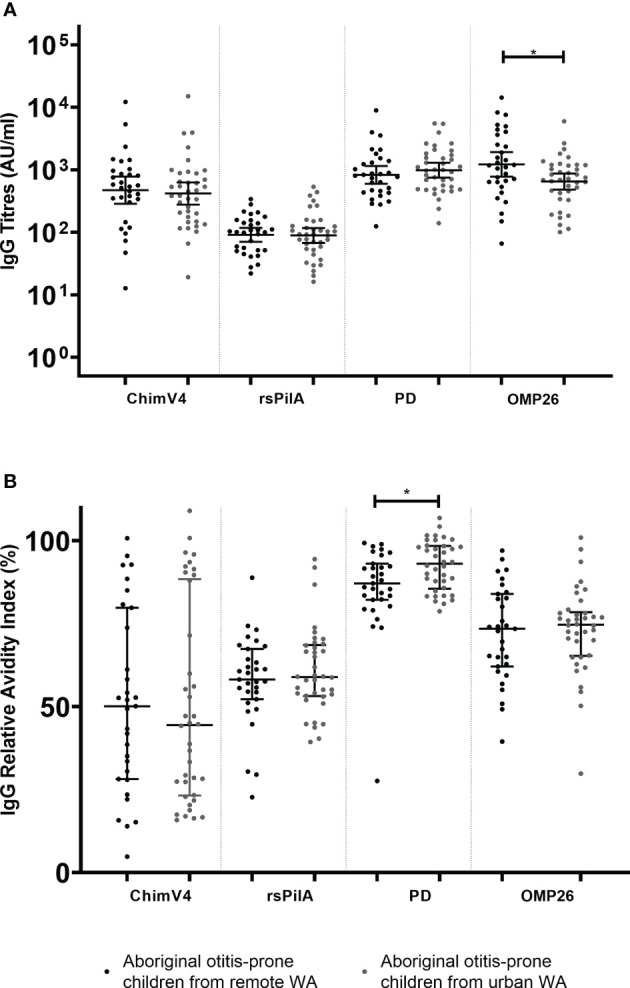
Comparison of serum IgG titres and relative avidity indices (RAI)between Aboriginal otitis-prone children living in remote and urban Western Australia. **(A)** Comparison of serum IgG titres between remote and urban Aboriginal otitis-prone children. Levels of serum IgG against NTHi proteins are presented for each individual child, with the horizontal bars depicting the GMC ± 95%CI (not age adjusted). Statistical analyses were conducted on the log transformed GMC data, adjusted for age. **(B)** Comparison of serum IgG RAI between remote and urban Aboriginal otitis-prone children. RAI serum IgG against NTHi proteins are presented for each individual child, with the horizontal bars depicting the median ±Interquartile Range. ChimV4, chimeric protein V4 (rsPilA+ protein 5), rsPilA,recombinant soluble PilA; PD, Protein D; OMP26, outer membrane protein 26. *P ≤ 0.05.

This cohort contains Aboriginal otitis-prone children from both urban and remote areas (**Table 1**). IgG titres were compared between these groups. Antibody titres specific for rsPilA, ChimV4 and PD were similar between Aboriginal otitis-prone children who lived in remote areas and those who lived in urban areas (GMCs: rsPilA = 91.1 vs 89.0; ChimV4 = 471.6 vs 415.5 and PD = 828.3 vs 984.6; p> 0.05) (**Figure 2A**). However, anti-OMP26 IgG titres were two-fold higher in children from remote areas when compared to those from urban areas (GMCs: 1188.2 vs 648.5; p=0.012) (**Figure 2A**). When antibody avidity was assessed, the RAIs of IgG specific for rsPilA, ChimV4 and OMP26 were similar in all children regardless of whether they lived remotely or in an urban area (median RAI remote versus urban: 58% vs 59%, 50% vs 44%, and 73% vs 75% respectively) (**Figure 2B**). However, children from urban areas had higher RAIs to PD compared to those from remote areas (93% versus 87% respectively, p<0.05).

## Discussion

In this cross-sectional study of 173 Australian Aboriginal and non-Aboriginal children, we examined naturally acquired serum IgG titres and avidity to potential NTHi vaccine antigens: rsPilA, ChimV4 and OMP26. Avidity indices were also measured for PD-specific IgG, for which PD IgG titres have been previously reported for this cohort (Thornton et al., 2017b). We found that naturally induced IgG titres to all NTHi antigens were similar between non-Aboriginal otitis-prone and non-otitis-prone children. In contrast, serum IgG titres against rsPilA and ChimV4 were significantly lower in Australian Aboriginal otitis-prone children compared to non-Aboriginal children regardless of diagnoses. IgG titres against OMP26 were similar for all groups. Notably, there were no differences in the avidity of antigen-specific IgG for rsPilA, ChimV4 or PD, however non-otitis-prone children had lower avidity IgG to OMP26 when compared to otitis-prone children. Weak relationships were observed between IgG titres, avidity indices and age for any antigen.

Australian Aboriginal children have been shown to develop chronic OM in infancy and this is associated with early, frequent and diverse NTHi carriage (Leach et al., 1994; Wenxing et al., 2012; Pickering et al., 2014). Despite repeated exposure to NTHi, serum IgG titres specific for rsPilA and ChimV4 were 50% lower in Aboriginal children. Interestingly, no differences were observed for IgG titres against OMP26. These antigen-specific differences are consistent with previous data from this cohort, which showed lower IgG titres to PD but similar levels for outer membrane proteins P4 and P6 in Aboriginal children when compared to both non-Aboriginal otitis-prone and non-otitis-prone children (Thornton et al., 2017b). Previous studies have shown that *in vitro* and *in vivo* OM models antibodies specific for rsPilA can prevent biofilm formation and disrupt established biofilms (Novotny et al., 2015). This specific reduction in IgG titres to rsPilA epitopes (which are also included in ChimV4) may leave children more susceptible to chronic and recurrent infections due to their essential role in the formation and maintenance of biofilms (Jurcisek et al., 2007). Data from murine models have also shown that a certain threshold of PilA antibodies are required to prevent NTHi nasopharyngeal colonisation (Ysebaert et al., 2019). Currently, it is unknown what antibody titres are needed to disrupt biofilms at the mucosal surface *in vivo*. This raises the possibility that a vaccine containing PilA such as, NTHi-10-AS01E, which is immunogenic in adults (Wilkinson et al., 2019; Van Damme et al., 2019), may have a role in preventing NTHi OM in Aboriginal children.

The reduced IgG titres to rsPilA and ChimV4 observed in Aboriginal otitis-prone children compared to non-Aboriginal otitis-prone children may be due to earlier and more dense NTHi colonisation (Leach et al., 1994; Wenxing et al., 2012) and colonisation with multiple strains in this population (Pickering et al., 2014). This early colonisation during development of tolerance to self-antigens may mistakenly also result in tolerance to NTHi antigens (Renz et al., 2018). An alternative explanation may be that these children are able to produce antibody, but these are depleted due to high pathogen exposure; this would require a longitudinal study with concurrent microbiological specimens to assess. Interestingly, unlike in our previous work for PD, no differences were observed in the rsPilA and ChimV4 antibody titres between non-Aboriginal children with and without OM. Previous data by Novotny et al. demonstrated that proportionately, otitis-prone children in the USA had reduced antibody titres to the protective epitopes of PilA compared to non-otitis-prone children (Novotny et al., 2009). This may suggest that in our cohort, while IgG titres between non-Aboriginal otitis-prone and non-otitis-prone children are similar, the proportion of effective antibody may be different. Together these data suggest that vaccination strategies to induce higher IgG titres against protective epitopes of PilA could be considered in these populations.

When we assessed relationships for IgG titres between different antigens to understand antibody development, IgG against PD (previously measured) (Thornton et al., 2017b) positively correlated with anti-rsPilA and anti-ChimV4, but not anti-OMP26 IgG titres. This may indicate that the biological mechanisms leading to reduced antigen-specific IgG titres for rsPilA, ChimV4 and PD are similar. Understanding these immune mechanisms will be crucial to the development of vaccines that can boost protective antibody levels in Aboriginal children.

Although Aboriginal children had reduced serum IgG titres, the relative avidity of PD, rsPilA and ChimV4 IgG were similar between the groups, suggesting that otitis-prone children can produce high-quality antibody. Despite this, the lower antibody titres produced by Aboriginal otitis-prone children may result in levels below protective thresholds despite being high-quality. Previous studies assessing vaccine induced antibody against *Haemophilus influenzae* type B and *Streptococcus pneumoniae*, show that higher avidity antibodies are more protective through improved bactericidal activity or opsonisation (Schlesinger et al., 1992; McGhee et al., 1999). Together these data suggest that reduced amounts of these high avidity IgG may contribute to the chronicity of OM observed in this population, and this warrants further investigation.

OMP26 IgG titres were similar in all children within the cohort, however, non-otitis-prone children had significantly reduced RAI in comparison to otitis-prone children. Only one study has reported OMP26 IgG in sera of otitis-prone children, demonstrating titres to be higher than in non-otitis prone children (Ysebaert et al., 2019). When assessed for bactericidal activity, OMP26 IgG was not bactericidal to NTHi in either group (Pichichero et al., 2010; Khan et al., 2012). However, rat studies have shown that vaccination with OMP26 produces bactericidal antibodies (Ercoli et al., 2015; Singh et al., 2020). In addition, previous animal work has shown that responses to OMP26, and the subsequent protection afforded by these, differ based on natural infection or vaccination (Kyd et al., 2003). Therefore, while natural antibody responses highlight important antigens, they only form part of the pre-clinical data needed to support moving vaccine antigens into clinical trials and this is a limitation of our study.

Similar to previous reports in this cohort, no relationships were observed between age and IgG titres, nor was RAI related to age. This is likely due to the older age range included in this study and it would be expected that all have been exposed to NTHi. The lack of microbiological data in this cohort is another limitation, however several studies have demonstrated that PD IgG titres in healthy children peak by approximately 3 years of age and then stabilise (Hua et al., 2016; Igor et al., 2016). Together these data suggest that IgG titres in these children have reached their peak production from natural exposure and further increases would need to be elicited by carefully developed vaccines.

Historically, research into OM in Australian Aboriginal children has concentrated in remote communities even though most Aboriginal children live in urban areas (Statistics ABo, 2018). The focus on children living in remote areas was based on the assumption that children from remote communities have more severe disease than those in urban areas. However, it has been shown recently that Australian Aboriginal children from urban areas in Western Australia have similar rates of OM as those reported for remote communities (Swift et al., 2020); suggesting that environmental risk factors alone may not lead to increased susceptibility of Australian Aboriginal children to OM. This is supported by our data showing similar IgG titres for 3/4 NTHi antigens, with similar quality, being observed between locations. OMP26 specific IgG was reduced in children from urban areas in comparison to children from remote areas and this may reflect differences in levels of NTHi exposure (and NTHi strain diversity (Pickering et al., 2014) as well as environmental risk factors. These data suggest a vaccine containing PD, rsPilA and ChimV4 may benefit all Australian Aboriginal children.

In summary, we have shown that Australian Aboriginal children can produce high quality IgG to the important NTHi antigens, rsPilA and ChimV4, but at reduced levels. Boosting antibody titres through vaccination strategies using these antigens is therefore warranted to impact the chronicity and high burden of OM in this population.

## Data Availability Statement

The raw data supporting the conclusions of this article will be made available by the authors, without undue reservation.

## Ethics Statement

The studies involving human participants were reviewed and approved by Princess Margaret Hospital for Children, Armadale-Kelmscott Memorial Hospital Ethics Commitee, Osborne Park Hospital Ethics Committee, the Western Australian Aboriginal Health Ethics Committee, and Aboriginal community-controlled health services in the Kimberley and Eastern Goldfields regions.

## Author contributions

SC and KC developed and conducted serological testing. SC analysed antibody data and drafted the manuscript. RT, SV, PR and HC were responsible for sample collection. AC, LN and LB provided proteins for assays and expert advice during assay development and manuscript preparation. ES, LK, RT, and PR oversaw sample selection, experimental conduct, analysis, data interpretation and manuscript preparation. PR, RT and HC obtained funding for study initiation and recruitment. All authors contributed to the article and approved the submitted version.

## Funding

This work was supported by funding from the Wesfarmers Centre of Vaccines and Infectious Diseases, Telethon Kids Institute, a Western Australia, Department of Health Near Miss Award (awarded to RT), and the Perth Children's Hospital Foundation. This study was supported, in part, by NIDCD/NIH R01 DC003915. This cohort was established thanks to funding by the Passe and Williams Foundation. SC was supported by a Research Training Program stipend and Wesfarmers Centre of Vaccines and Infectious Diseases top up scholarship.

## Conflict of Interest

LB is an inventor of technology related to PilA-derived immunogens that is licensed to GlaxoSmithKline Biologicals. PR reports receiving funds to his institution from GlaxoSmithKline for investigator led research and participation in vaccine scientific advisory boards outside the submitted work.

The remaining authors declare that the research was conducted in the absence of any commercial or financial relationships that could be construed as a potential conflict of interest.

## Publisher’s Note

All claims expressed in this article are solely those of the authors and do not necessarily represent those of their affiliated organizations, or those of the publisher, the editors and the reviewers. Any product that may be evaluated in this article, or claim that may be made by its manufacturer, is not guaranteed or endorsed by the publisher.
