# Supplementary material for: Australian Aboriginal Otitis-Prone Children Produce High-Quality Serum IgG to Putative Nontypeable Haemophilus influenzae Vaccine Antigens at Lower Titres Compared to Non-Aboriginal Children
Source: Front Cell Infect Microbiol. 2022 Apr 7;12:767083. doi: 10.3389/fcimb.2022.767083 (PMC9022120; doi:10.3389/fcimb.2022.767083)
Supplement: Supplementary file 1 [file Image_1.pdf]

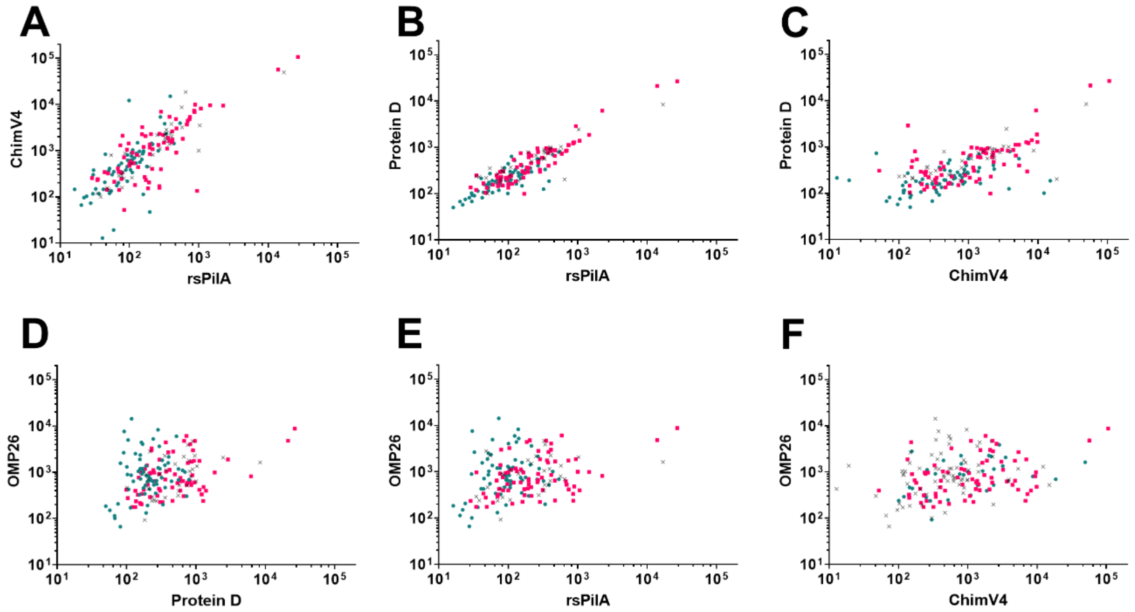

Supplementary Figure 1: Correlation of serum IgG titres between different NTHi antigens in Aboriginal otitis-prone, non-Aboriginal otitis-prone and non-otitis prone children. (A) comparison of serum IgG titres between rsPilA and ChimV4, R=0.806, p< 0.001. (B) Comparison of serum IgG titres between rsPilA and PD, R= 0.898, p<0.001. (C) Comparison of serum IgG titres between ChimV4 and PD, R= 0.662, p<0.001. (D) Comparison of serum IgG titres between OMP26 and PD, R=0.306, <0.001. (E) Comparison of serum IgG titres between OMP26 and rsPilA, R= 0.301, p<0.001. (F) Comparison of serum IgG titres between OMP26 and ChimV4, R=0.302, p< 0.001. black cross= non-otitis-prone children; pink square = non-Aboriginal otitis-prone children; teal circle = Aboriginal otitis-prone children. ChimV4, chimeric protein V4 (rsPilA+ protein 5), rsPilA, recombinant soluble PilA; PD, Protein D; OMP26, outer membrane protein 26.
